# Generation of FX ^−/−^ and Gmds ^−/−^ CHOZN host cell lines for the production of afucosylated therapeutic antibodies

**DOI:** 10.1002/btpr.3061

**Published:** 2020-08-26

**Authors:** Weiyi Liu, Roshan Padmashali, Omar Quintero Monzon, Dianna Lundberg, Shan Jin, Brian Dwyer, Yun‐Jung Lee, Anisha Korde, Sophia Park, Clark Pan, Bohong Zhang

**Affiliations:** ^1^ Rare Disease Unit Takeda Pharmaceutical Company Limited Cambridge Massachusetts USA

## Abstract

Antibody‐dependent cellular cytotoxicity (ADCC) is the primary mechanism of actions for several marketed therapeutic antibodies (mAbs) and for many more in clinical trials. The ADCC efficacy is highly dependent on the ability of therapeutic mAbs to recruit effector cells such as **n**atural **k**iller cells, which induce the apoptosis of targeted cells. The recruitment of effector cells by mAbs is negatively affected by fucose modification of N‐Glycans on the Fc; thus, utilization of afucosylated mAbs has been a trend for enhanced ADCC therapeutics. Most of afucosylated mAbs in clinical or commercial manufacturing were produced from Fut8^−/−^ Chinese hamster ovary cells (CHO) host cells, generally generating low yields compared to wildtype CHO host. This study details the generation and characterization of two engineered CHOZN® cell lines, in which the enzyme involved in guanosine diphosphate (GDP)‐fucose synthesis, GDP mannose‐4,6‐dehydratase (Gmds) and GDP‐L‐fucose synthase (FX), was knocked out. The top host cell lines for each of the knockouts, FX−/− and Gmds−/−, were selected based on growth robustness, bulk MSX selection tolerance, production titer, fucosylation level, and cell stability. We tested the production of two proprietary IgG1 mAbs in the engineered host cells, and found that the titers were comparable to CHOZN® cells. The mAbs generated from either KO cell line exhibited loss of fucose modification, leading to significantly boosted FcγRIIIa binding and ADCC effects. Our data demonstrated that both FX−/− and Gmds−/− host cells could replace Fut8−/− CHO cells for clinical manufacturing of antibody therapeutics.

## 1 INTRODUCTION

Therapeutic mAbs have been developed and widely used for the treatment of multiple diseases, including autoimmune diseases and cancer. For some marketed oncology mAbs such as Trastuzumab, Rituximab, and Ipilimumab, the primary mechanism of actions could be attributed to antibody‐dependent cellular cytotoxicity (ADCC), a process that therapeutic marketed therapeutic antibodies (mAbs) bind to specific targeted cells and recruit effector cells, which induce the apoptosis of targeted cells.^1^, ^2^, ^3^, ^4^ The ADCC effect is highly dependent on the ability of therapeutic mAbs to recruit effector cells such as Natural Killer (NK) cells.^5^, ^6^ The recruitment of effector cells by mAbs is determined mainly by glycan‐glycan interaction between N‐glycosylations on the Fc domain of mAbs and FcγRIIIa (CD16a) on effector cells.^7^ Based on the crystal structure, afucosylated glycans at Asn297 of IgG1 recognize the glycans at Asn162 of FcγRIIIa through Hydrogen bond and van der Waals interactions.^8^, ^9^ If the IgG1 glycans were fucosylated, the presence of fucose breaks key polar interactions between the glycans and induces steric hindrance, causing a shift away from the receptor's glycans.^8^, ^10^ The utilization of afucosylated mAbs has been a trend for enhanced ADCC therapeutics. Some recently marketed ADCC mAbs such as Benralizumab targeting IL5R, Obinutuzumab targeting CD20, and Mogamulizumab targeting CCR4, were all designed and manufactured as afucosylated mAbs.^1^, ^11^, ^12^

For the clinical and commercial manufacturing of afucosylated mAbs, multiple strategies have been applied. Mostly expressed in CHO cells, mAbs are glycosylated at the endoplasmic reticulum, and the glycans are further modified at the golgi apparatus. In the Golgi glycans are fucosylated by fucosyltransferase 8 (Fut8), the only expressed fucose‐transferase in CHO cells.^13^, ^14^ So far, majorities of afucosylated mAbs in clinical or commercial manufacturing were produced from Fut8^−/−^ CHO host cells, which usually have retarded cell growth, less viable cell densities and lower production titers.^15^, ^16^ The addition of Fut8 chemical inhibitor 2F‐Peracetyl‐Fucose into the cell culture medium to generate afucosylated mAbs is another approach. However, the batch‐to‐batch consistency in both productivity and afucosylation levels in large manufacturing bioreactors need further evaluation.^17^ Glycoengineering of host cell lines by overexpression of N‐acetylglucosaminyl transferase (GNTIII) led to the modification of N‐glycans to bisecting GlcNAc glycans which dramatically reduce the addition of fucose.^18^

An alternative approach is to modify host CHO cells through engineering the de novo synthesis pathway of GDP‐fucose. GDP‐fucose, the substrate of protein fucosylation, is synthesized in the cell cytosol from GDP‐mannose, through two‐step biochemical reactions catalyzed by the enzymes Gmds and FX, and transported into the Golgi apparatus by Slc35C1.^19^, ^20^ Knockout (KO) of Gmds based on homologous recombination in CHO/DG44 cells led to a complete depletion of intracellular GDP‐fucose and afucosylation of expressed mAbs.^19^ Likewise, KO of FX with CRISPR‐Cas9 in CHO‐K1 cells led to completely afucosylated mAbs.^15^ The study suggested that there might be three alleles of FX in the CHO genome, and two guide RNAs were used to get a functional FX^−/−^ host cell line, which makes cell engineering on FX gene complicated. In addition, overexpression of enzyme RMD that converts GDP‐mannose, the Gmds substrate, to a metabolically dead‐end product also blocks GDP‐fucose synthesis.^21^ The effects of the dead‐end metabolites on CHO production, the stability of RMD expression and downstream removal of the RMD enzyme from drug substance have to be taken into account. Inactivation of the GDP‐mannose transporter (Slc35C1) also can be used to produce of afucosylated mAbs.^20^ In this study, we applied the CRISPR‐Cas9 engineering tool in the industry widely used CHO cells of glutathione synthetase genomic locus disrupted by Zinc‐Finger nuclease (CHOZN) (GS^−/−^ CHOK1) cells, and detailed the generation, characterization and product quality evaluation of FX^−/−^ and Gmds^−/−^ CHOZN cells, paving the way for next generation of host cell lines for the production of afucosylated therapeutic mAbs.

## 2 MATERIALS AND METHODS

### 2.1 Cell culture and engineering

CHOZN® cells (GS^−/−^ CHOK1, Sigma) were passaged regularly in EX‐Cell CD CHO Fusion medium (Sigma 14365C) containing 6 mM Glutamine. Per gRNA transfection, 1ug TrueCut Cas9 V2 proteins (Thermo Fisher A36496), 200 ng CRISPR IVT gRNA (Table 1), and 10 pmol ss‐Oligos (Table 1) in 10ul reaction volume were electroporated into 3.0E + 6 cells using NEON electroporator, following the optimized parameters of 1,600 V pulse voltage, 10 ms each pulse for three pulses. In 72 hr posttransfection, a portion of transfection cells were processed for the estimation of gene editing efficiency using GeneArt genomic cleavage detection kit (Thermo Fisher A24372). The rest of cells were cultured to expand for stable pool generation. The pools of highest editing efficiency were single cell sorted into 10 plates of 96‐well plates. Once confluent, the clones were consolidated to one 96‐well plate and gRNA‐targeted genomic regions were polymerase chain reaction (PCR) amplified (Table 1) and sequenced with next generation sequencing.

**TABLE 1:** Sequences of gRNAs, ss‐Oligos, and primers

gRNAs			
CRISPR name	Sequence (5′‐3′)	PAM	Location
Gmds.1	CGATCCAGTTCATTTAATAC	GGG	Exon1
Gmds.2	GCAGAATTCCTGCTGGAGAA	AGG	Exon1
Gmds.3	ACATTTATATAAGAATCCAC	AGG	Exon1
FX.1	CCAGGAGGATCCTAGTGACA	AGG	Exon2
FX.2	TGGGTGAGCCCCAGGGATCC	AGG	Exon1
FX.3	GTCCAGAGCCCCCTGTCACT	AGG	Exon2
ssOligos			
CRISPR name	Ss‐Oligo sequence (5′‐3′)		
Gmds.1	gOFacttcatgtttccttcaatatgagcctgtggattcttatataaatgttcaattcgacctCAattaaatgaactggatcgccgtacaattccatgFFc		
Gmds.2	aFEgtggcgctcatcacgggcatcaccggccaggatggctcatacttggcagaattcctgctgTagaaaggatacgaggtgagtgactcagtgctccZEg		
Gmds.3	tOFtggaattgtacggcgatccagttcatttaatacaggtcgaattgaacatttatataagaatTGacaggctcatattgaaggaagtaagtaatttZOt		
FX.1	tEEtggaaccctgcgcaggtgcagcaacaatgggtgagccccagggatccaggaggatcctagtgTAagggggctctggac‐tggtgggcagagctatOOa		
FX.2	cEEaagtagctcttggactggtggaaccctgcgcaggtgcagcaacaatgggtgagccccagTgatccaggaggatcctagtgacagggggctctggFOt		
FX.3	Same as FX.1		
	F: A‐Phosphorothioate, O: C‐Phosphorothioate, E: G‐Phosphorothioate, Z: T‐Phosphorothioate		
Primers			
FX	5′‐3′ sequences		
FX_gcdF1	CCTAGTAGGCTCATGACTTGGAACCAAGC		
FX_gcdF2	CAGTCGTAGGGATGAGATGTATC		
FX_gcdR1	GGTTACTGATCTGGCTGTGTTTGCTGCC		
FX_gcdR2	GCAGTAGCCCTCCCTGAGTCTGGGTCCGC		
Gmds	5′‐3′ sequences		
Gmds_gcdF1	GCAGTACAAACCCTGGATAAGGTCAGAAGC		
Gmds_gcdF2	GCAGTGTGCACACAGGCAGTAGTGAGG		
Gmds_gcdR1	GCCATCTTGGTTTGATTATTAGAGTG		
Gmds_gcdR2	CTTTAGTCAGCCTTCTTCAGTCCAGG		

Abbreviation: Gmds, GDP mannose‐4,6‐dehydratase.

### 2.2 FITC‐LCA staining

Cells (5.0E + 5/well) were cultured in suspension in 96‐deep well plates for 36 hr in 1 ml culture medium, with or without addition of 10 mM Fucose. A total of 2.0E + 5 cells per well were spin down and incubated with FITC conjugated LCA (Vector laboratory, FL‐1041) at a final concentration of 2ug/ml for 30 min on ice. Cells were washed and analyzed at an attune analytic flow cytometer, and data was analyzed with Flowjo.

### 2.3 Bulk transfection and MSX selection

Following the electroporation procedure, linearized plasmids (10ug) carrying GS and mAb expression cassettes was electroporated into 2.0E + 7 cells, and cells were seeded into T‐25 cm2 flask with 5 mL selection medium (EX‐CELL® CD CHO Fusion+6uM methionine sulfoximine [MSX]). 24 hr posttransfection, the cells were transferred to a sterile 15 mL conical tube and centrifuged at 1000 RPM. The cell pellet was resuspended in 10 mL corresponding selection medium, transferred to a T‐75 cm2 cell culture flask, and incubated at 36.5C 5% CO2, in 80% humidified incubator. At 7 day, a complete 10 ml medium exchange was performed. At day 13–18, once the viability reached over 50%, cells were transferred into E125 flask at a minimal viable cell density of 0.2 × 10e6 cells/ml, to shake at 125RPM, 36.5°C/5% CO2. After recovered from selection, the cells were routinely passaged in the selection medium.

### 2.4 Western blot

A total of 1.0E + 8 CHOZN cells were lysed in 1 ml radioimmunoprecipitation assay buffer (RIPA) buffer (Thermo Fisher Cat# 89901) including.

Protease and phosphatase inhibitor (Pierce Cat# A32961). Protein samples (300ug) mixed with 4x Bolt LDS sample buffer and 10X Bolt reducing buffer were boiled for 10 min at 70°C and run for 4%–12% SDS‐PAGE gel. Proteins was transferred to PVDF membrane using iBlot®2 Dry Blotting System. Primary antibodies are rabbit anti‐Gmds Ab (Abcam 97,630, 1:500), mouse Anti‐GAPDH Ab (Sigma M2, 1:10000), and rabbit polyclonal antibody anti‐FX (GeneTex Cat#101663, 1:2000). Secondary antibodies are goat anti‐rabbit IgG (H + L) (IRDye® 800CW P/N 925–32,211, 1:10000) and goat anti‐mouse IgG (H + L) (IRDye® 680RD P/N 925–68,070, 1:10000). PVDF membranes were imaged using Li‐Cor Imaging system.

### 2.5 Fed‐batch production and mAb purification

Following Sigma CHOZN fed‐batch protocol, inoculate 30 ml of cells at an initial starting cell density of 0.3E + 6 cells/ml in EX‐CELL Advanced CHO fed‐batch medium (14366C) in a 125 ml Erlenmeyer shake flask. From day 4 postinoculation, every other day proper amount of glucose (G8769) and 2 ml Feed1 medium were feed to make the final concentration to 7‐8 g/L of glucose. Cells were counted and titers were measured using Octet for day7, day11, and day14. The day14 conditional medium was used for affinity chromatography with HiTrap MabSelect SuRe prepacked column (GE 11003494), and mAbs were eluted with Elution buffer (50 mM Acetic Acid, pH 3.5).

### 2.6 Glycan analysis

To characterize mAb‐bound N‐glycans, purified mAbs were re‐formulated in 50 mM Bis/Tris pH 6 buffer to a concentration over 2 mg/ml by diafiltration using microcon 30KDa spin filters (cat. No.: MRCF0R030, Millipore). N‐glycans were then released and processed for Gly‐Q analysis, using the Gly‐X™ kit (Cat. No.: GX96‐IQ, Prozyme Inc), according to the manufacturer's instructions. Briefly, mAbs were subjected to heat denaturation in reduced conditions, followed by N‐glycans release using Prozyme's proprietary fast PNGase F protocol. Glycans were then rapidly labeled with a proprietary negatively charged fluorescent reagent (InstantQ). The labeled N‐Glycans, free protein, and excess labeling agent were then subjected to clean up on plate‐based columns that absorb sugars selectively. The enriched labeled glycans were then eluted from the columns and analyzed by CE coupled with fluorometric detection. To monitor separation quality and to report relative glycan migration, a prelabeled glucose size ladder was run interspersed between samples at evenly spaced intervals. Glycan's migration was reported as GU, based on the two nearest migrating glucose markers in the ladder. To assign structures to unknown glycans, GU migrations were compared to those of known glycan structures using the manufacturer's provided analysis software.

### 2.7 LC‐FLR‐MS/MS for released N‐glycan analysis

Waters UPLC with Fluorescence detector was used as LC‐FLR system. Waters ACQUITY UPLC Glycan BEH Amide 130 A (1.7 um, 2.1 × 150 mm) column was used for LC and the column temperature was set as 55°C. Acetonitrile (100%) was used as mobile phase A and freshly prepared 100 mM Ammonium formate in water was used as mobile phase B. A shallow gradient of 30%–40% of phase B from 6 min to 42 min was used for separation. Weak wash was performed with 600 ul of 25 mM Ammonium formate in 25% water and 75% acetonitrile, and strong wash with 200 ul of 100 mM Ammonium formate in water. Injection volume was 15 ul for each sample. Excitation at 265 nm and emission at 425 nm were applied as fluorescence wavelengths, and fluorescence sampling rate was set as 2 Hz. Thermo Fischer Q ExactiveP™P Plus mass spectrometer was applied for m/z detection. MS acquisition mode was set as positive full MS/ddMS2, full MS scan resolution was set as 17,500, and AGC target was set as 3e6, with 100 ms of Maximum injection time (IT). Scan range was 700–3,500 m/z, the dd‐MS2/dd‐secondary ion mass spectrometry (SIM) scan resolution was set as 17,500, and AGC target was 1e5, with 50 ms of Maximum IT. TopN was set as six, and the isolation window was 2 m/z and (N)CE was 27.

### 2.8 Biacore binding assay

Following GE Biacore assay handbook, the mAbs used for testing were diluted into 5ug/ml in Sodium Acetate buffer (pH 5.0), and were immobilized to CM5 Chip (GE, BR100530). FcγRIIIa (R&D system, 4,325‐FC‐050) and FcγRIa (R&D system, 1,257‐FC‐050) were serial diluted with HBS‐EP + (GE, BR100827). The association is 300 s and dissociation is 600 s, at the flow rate of 30 uL/min. Multi‐cycle kinetics were performed with the regeneration buffer (10 mM phosphate and 500 mM NaCl, pH 2.5), for 10s at the rate of 30 μl/ml. Binding kinetics and KD(M), were determined using Languir 1:1 binding model and compared for different cell lines.

### 2.9 ADCC assay

ADCC target cell line was a multiple myeloma‐derived suspension cell line with high antigen expression recognized by mAb2. The target cells were seeded at a density of 15,000 cells/well in a 96‐well U‐bottom plate in a 50uL volume of ADCC assay buffer (serum free RPMI‐1640 media +1%BSA + 2 mM L‐Glutamine). Cells were then incubated with serial dilutions of test antibodies for 30 min at 37°C in 50uL of ADCC buffer. Target cells were finally exposed to purified NK cells in 50 ul ADCC buffer at a ratio of 3:1 (effector: target) to induce ADCC mediated cell lysis. The ADCC lysis endpoint was measured after 4 hr in culture at 37°C by collecting 100 ul of supernatant from each well. Cell lysis was measured by lactate dehydrogenase (LDH) release with a Cytotoxicity Detection KitPLUS from Roche according to manufacturer's instructions. Control wells included wells for measuring spontaneous target cell lysis (without NK cells), and complete cell lysis (target cells lysed with 10 ul of cell lysis solution provided in the LDH kit). NK cells were purified from fresh whole human blood with human NK cell isolation kit (cat#130–092‐657) from Miltenyi Biotec according to protocol provided in the kit.

ADCC % cell killing was calculated as:

$$\left(\left[\text{Sample well}\right]\mathrm{Abs}\;49\text{0nm}–\left(\text{Sample well with}\;\mathrm{no}\;\text{antibody}\right)\mathrm{Abs}\;490\;\mathrm{nm}\right)\;]\times \;100/\left(\left[\text{Complete cell lysis}\right]\mathrm{Abs}\;49\text{0nm}\;–\left(\text{Spontaneous target cell lysis with}\;\mathrm{no}\;\mathrm{NK}\;\text{cells}\right)\mathrm{Abs}\;490\;\mathrm{nm}]\right)$$

## 3 RESULTS

### 3.1 Generation of FX ^−/−^ and Gmds^−/−^ CHOZN host cell lines using CRISPR/Cas9 system

**FIGURE 1:**
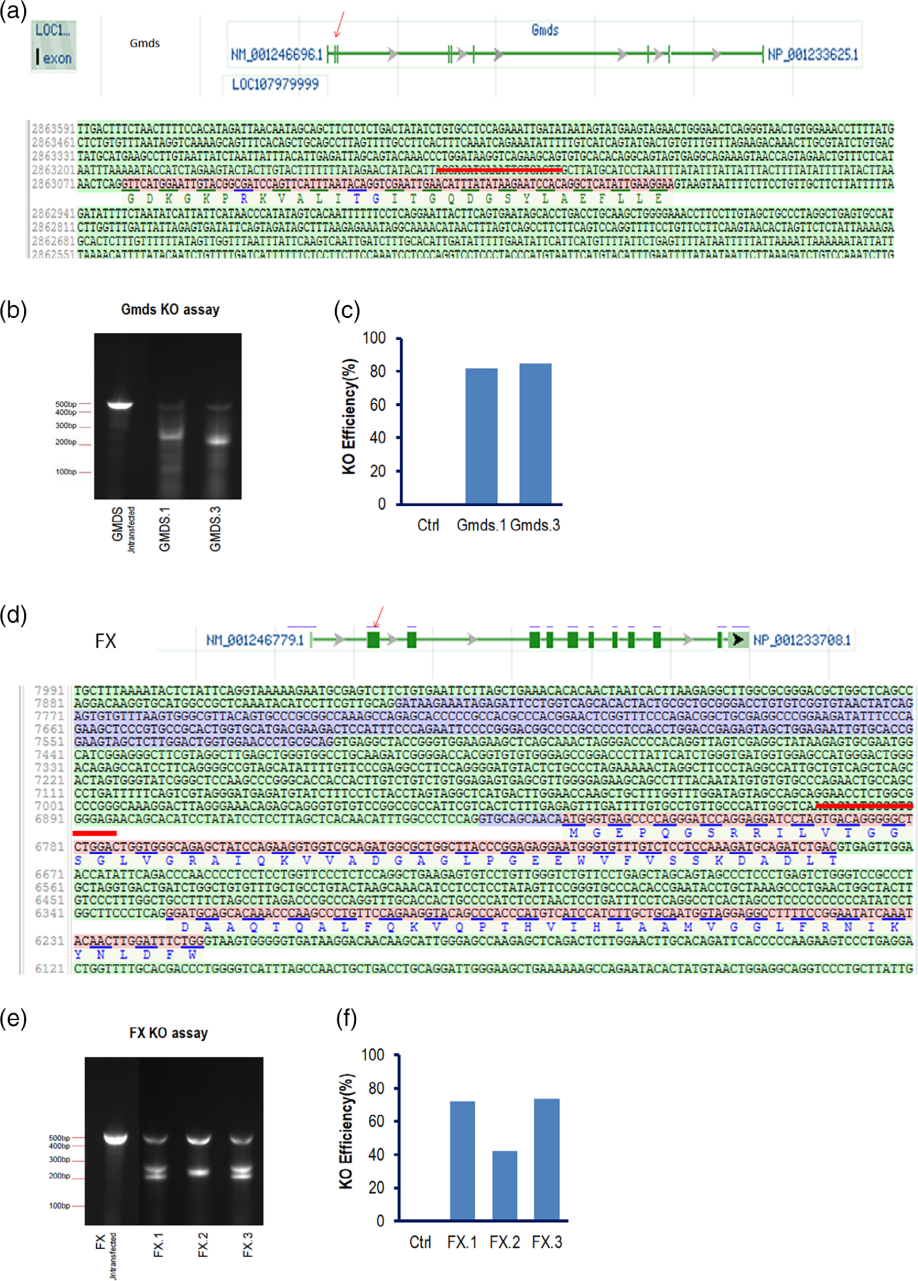
gRNA design and efficiency tests. (a), the exon schematic of Gmds in CHO genome. The red arrow point to the exon where gRNAs were targeting. The exon sequences were displayed and the red line highlighted Gmds.3‐recognized sequences. (b), agarose gel electrophoresis for genomic cleavage assay to examine editing efficiency of gRNA Gmds.1 and gRNA Gmds.3. The top ~500 bp PCR band was uncleavable (from unedited alleles) and the bottom ~250 bp band was cleaved from edited alleles. The ratio of top vs bottom bands represented the ratio of wildtype vs edited alleles. (c), calculated percentage of cells edited at Gmds locus. (d), the exon schematic of FX genome and the exon that gRNA FX.3 is targeting. (e), genomic cleavage assay for FX.1, FX.2, and FX.3. The top ~500 bp PCR band was un‐cleavable (from unedited alleles) and the bottom 200–300 bp bands represent edited alleles. (f), calculated percentage of cells edited at FX locus. Gmds, GDP mannose‐4,6‐dehydratase

The CHO cells used in the study are CHOZN® cells from Sigma, which has glutamine synthetase (GS) KO GS^−/−^ genotype, often give higher biologics productivity and are widely used in the industry for clinical cell line development. The genomic sequences of FX and Gmds in CHOZN background were referenced from the published genomic database of CHO‐K1 cells (CriGri‐PICR, GCF_003668045.1). To generate the KO cells by CRISPR/cas9 systems, three guide RNAs (gRNAs) were designed against first or second exon for each target (Table 1, Figure 1a,d showed genomic schematics). PCR amplification of the deoxyribonucleic acid (DNA) fragments covering gRNA‐targeted genomic loci of the CHOZN cells were sequenced (data not shown), and the targeting sequences were identical to published sequences from CHO‐K1 cells. For each target, we also synthesized three single‐strand oligonucleotides (ssOligos) that carried a stop codon and were homologous with the genomic loci where gRNAs are against (Table 1b). When cotransfected with gRNAs and Cas9 protein, ssOligos could enhance gene KO efficiency. The CHOZN pools were analyzed after transfection with gRNAs, ssOligos, and Cas9 protein. The cells transfected with a combo of gRNAs FX.1 and ssOligo FX.1 had 70% editing efficiency, a combo of gRNAs FX.2 and ssOligo FX.2 had 40% editing efficiency, and a combo of gRNAs FX.3 and ssOligo FX.3 had 72% editing efficiency. Similarly, the editing efficiency for a combo of gRNAs Gmds.1 and ssOligo Gmds.1 was 80%, and the editing efficiency for a combo of Gmds.3 guide ribonucleic acid (gRNA) and ssOligo Gmds.3 was 84% (Figure 1c,f). Gmds.2 gRNA was not synthesized successfully and dropped for further experiments. We performed single clone development from the pools that have been edited by FX.3 gRNA combo and Gmds.3 gRNA combo (gRNA binding genomic regions shown in Figure 1b,e).

**FIGURE 2:**
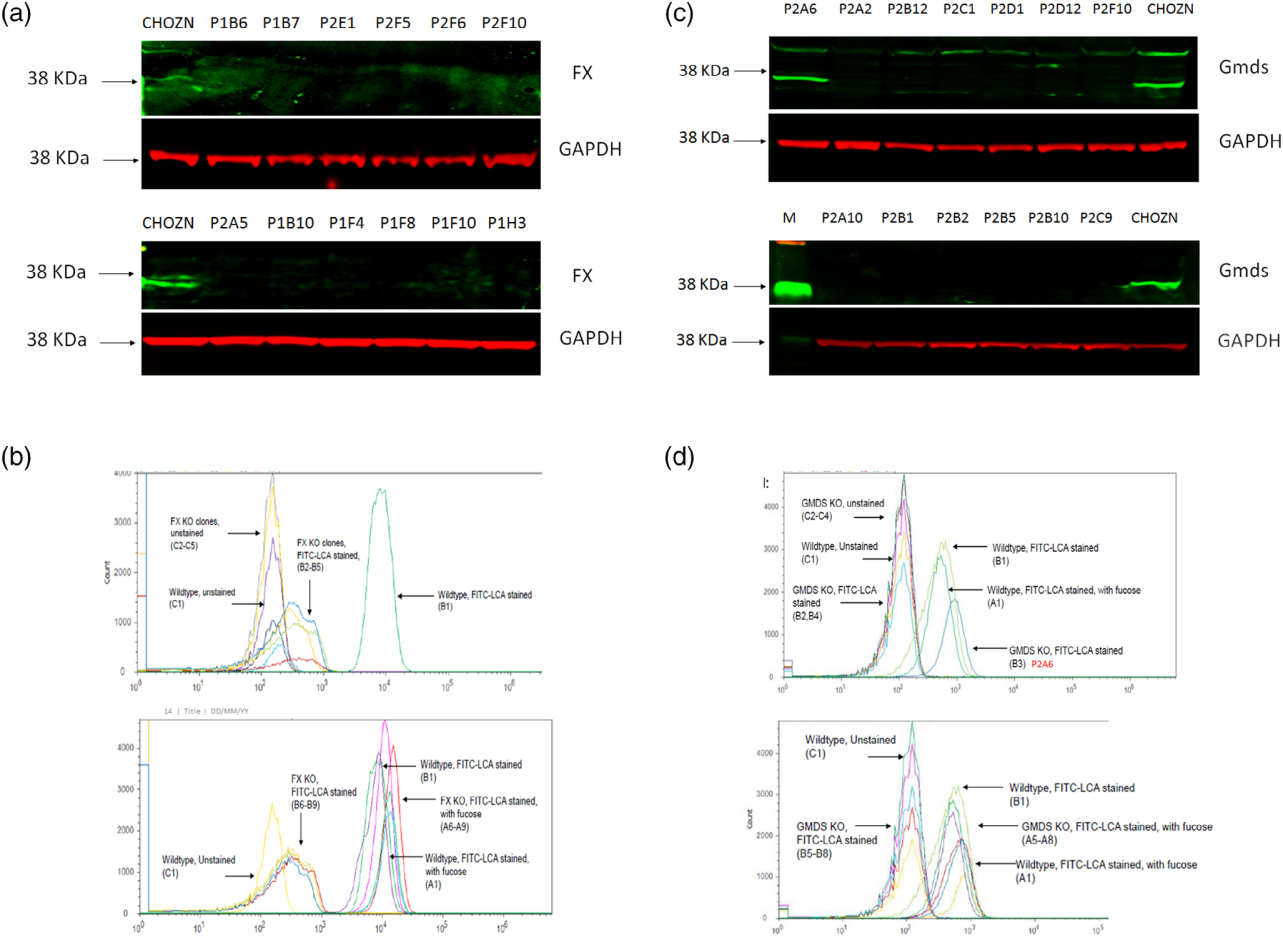
Characterization of Gmds^−/−^ CHOZN and FX^−/−^ CHOZN cells. (a), western blot of FX for expanded FX^−/−^ clones. (b) FITC‐LCA staining and flow cytometry analysis of FX^−/−^ clones, in the absence of fucose (top panel) or presence of fucose (bottom panel). (c), western blot of Gmds for expanded Gmds^−/−^ clones. (d) FITC‐LCA staining and flow cytometry analysis of Gmds^−/−^ clones, in the absence of fucose (top panel) or presence of fucose (bottom panel). Gmds, GDP mannose‐4,6‐dehydratase; LCA, lens culinaris agglutinin

For both Gmds and FX pools, a total of 96 clones were screened with PCR amplification of the gRNA‐targeted genomic region followed by next generation sequencing to examine sequence change. Top 12 clones carrying either nonsense deletions or insertions at both alleles (Table 2) for each gene were selected. Western blot results also confirmed that all the FX^−/−^ clones have lost the expression of the protein (Figure 2a), and 11 out of 12 Gmds^−/−^ clones have lost Gmds protein except clone P2A6 (Figure 2c). Lens culinaris agglutinin (LCA) recognizes α‐mannose glycan on cell surface glycoproteins and exhibits lower affinity to afucosylated glycan and increased affinity to fucosylated glycan. LCA‐Fluorescein isothiocyanate (FITC) staining demonstrated that except Gmds^−/−^ clone P2A6, all the 12 FX^−/−^ clones (Figure 2b) and 11 other Gmds^−/−^ clones (Figure 2d) had negative staining, indicative of disruption of the fucosylation pathway in those 23 clones. Clone Gmds^−/−^ P2A6 had a disrupted genotype but stained positive in LCA‐FITC, indicating it retains functional Gmds proteins, which could be caused by incorrect sample consolidation and therefore was dropped for further screening. When the 23 clones were cultured in the presence of fucose in medium, the LCA‐FITC staining turned to positive (Figure 2b,d), suggesting that the disruption of fucosylation pathway in those 23 clones could be rescued and that is consistent with previous studies that loss of de novo GDP‐fucose synthesis in CHO cells can be reversed by the addition of fucose in the medium.^15^, ^19^

**TABLE 2:** CRISPR/CAS9‐mediated sequence editing for the top FX−/− and Gmds−/− CHOZN clones

FX loci seq	TGAGCCCCAGGGATCCAGGAGGATCCTAGTG‐ACAGGGGGCTCTGGACTGGTGGGCAGAGCTATCCAGAAGGT
FXKO P1B3	TGAGCCCCAGGGATCCAGGAGGATCCTAGGG‐CAGGGGGCTCTGGACTGGTGGGCAGAGCTATCCAGAAGGT
FXKO P1B6	TGAGCCCCAGGGATCCAGGAGGATCCTAGT‐ACAGGGGGCTCTGGACTGGTGGGCAGAGCTATCCAGAAGGT
FXKO P1B7	TGAGCCCCAGGG‐ATCCAGAAGGT
FXKO P1C6	TGAGCCCCAGGGATCCAGGAGGATCCTAGTGGACAGGGGGCTCTGGACTGGTGGGCAGAGCTATCCAGAAGGT
FXKO P1F6	TGAGCCCCAGGGATCCAGGAGGATCCTAGT‐ACAGGGGGCTCTGGACTGGTGGGCAGAGCTATCCAGAAGGT
FXKO P2F5	TGAGCCCCAGGGATCCAGGAGGA‐TCTGGACTGGTGGGCAGAGCTATCCAGAAGGT
FXKO P2E1	TGAGCCC‐TGGTGGGCAGAGCTATCCAGAAGGT
FXKO P1B10	TGAGCCCC‐GGACTGGTGGGCAGAGCTATCCAGAAGGT
FXKO P1F4	TGAGCCCCAGGGATCCAGGAGGATCCTA‐GGGGCTCTGGACTGGTGGGCAGAGCTATCCAGAAGGT
FXKO P1F8	TGAGCCCCAGGGATCCAGGA‐GGACTGGTGGGCAGAGCTATCCAGAAGGT
FXKO P1F10	TGAGCCCCAGGGATCCAGGAGGATCCT‐GGGGGCTCTGGACTGGTGGGCAGAGCTATCCAGAAGGT
FXKO P1H3	TGAGCCCCAGGGATCCAGGAGG‐ CTCTGGACTGGTGGGCAGAGCTATCCAGAAGGT
FXKO P2A5	TGAGCCCCAGGGATCCAGGAGGATCC‐AGGGGGCTCTGGACTGGTGGGCAGAGCTATCCAGAAGGT

WT Gmds loci	GATCCAGTTCATTTAATACAGGTCGAATTGAACATTTATATAAGAATC‐CACAGGCTCATATTGAAGGAAGTAA
GmdsKO P2A6	GATCCAGTTCATTTAATACAGGTCGAATTGAACATTTATATAAGAATGA‐ACAGGCTCATATTGAAGGAAGTAA
GmdsKO P2A2	GATCCAGTTCATTTAATACAGGTCGAATTGAACATTTATATAAGAATGA‐ACAGGCTCATATTGAAGGAAGTAA
GmdsKO P2B12	GATCCAGTTCATTTAATACAGGTCGAATTGAACATTTATATAAGAATGA‐ACAGGCTCATATTGAAGGAAGTAA
GmdsKO P2C1	GATCCAGTTCATTTAATACAGGTCGAATTGAACATTTATATAAGAATGA‐ACAGGCTCATATTGAAGGAAGTAA
GmdsKO P2D1	GATCCAGTTCATTTAATACAGGTCGAATTGAACATTTATATAAGAATGA‐ACAGGCTCATATTGAAGGAAGTAA
GmdsKO P2D12	GATCCAGTTCATTTAATACAGGTCGAATTGAACATTTATATAAGAATGA‐ACAGGCTCATATTGAAGGAAGTAA
GmdsKO P2F10	GATCCAGTTCATTTAATACAGGTCGAATTGAACATTTATATAAGAATGA‐ACAGGCTCATATTGAAGGAAGTAA
GmdsKO P2A10	GATCCAGTTCATTTAATACAGGTCGAATTGAACATTTATATAAGA‐CAGGCTCATATTGAAGGAAGTAA
GmdsKO P2B1 50%	GATCCAGTTCATTTAATACAGGTCGAATTGAACATTTATATAAGAATCCCACAGGCTCATATTGAAGGAAGTAA
GmdsKO P2B1 50%	GATCCAGTTCATTTAATACAGGTCGAATTGAACATTTATATA‐CAGGCTCATATTGAAGGAAGTAA
GmdsKO P2B2	GATCCAGTTCATTTAATACAG‐AATTG‐ACAGGCTCATATTGAAGGAAGTAA
GmdsKO P2B5 50%	GATCCAGTTCATTTAATACAGGTCGAATTGAACATTTATATAAGAATCCCACAGGCTCATATTGAAGGAAGTAA
GmdsKO P2B5 50%	GATCCAGTTCATTTAATACAGGTC‐ACAGGCTCATATTGAAGGAAGTAA
GmdsKO P2B10	GATCCAGTTCATTTAATACAGGTCGAATTGAACATTTATATAAGAATTG‐ACAGGCTCATATTGAAGGAAGTAA
GmdsKO P2C9 50%	GATCCAGTTCATTTAATACAGGTCGAATTGAACATTTATATAAGAATTG‐ACAGGCTCATATTGAAGGAAGTAA
GmdsKO P2C9 50%	GATCCAGTTCATTTAATACAGGTCGAATTGAACATTTATATAAGAATCACACAGGCTCATATTGAAGGAAGTAA

Abbreviation: Gmds, GDP mannose‐4,6‐dehydratase.

### 3.2 Selection of lead FX ^−/−^ and Gmds^−/−^ CHOZN host cell lines

**FIGURE 3:**
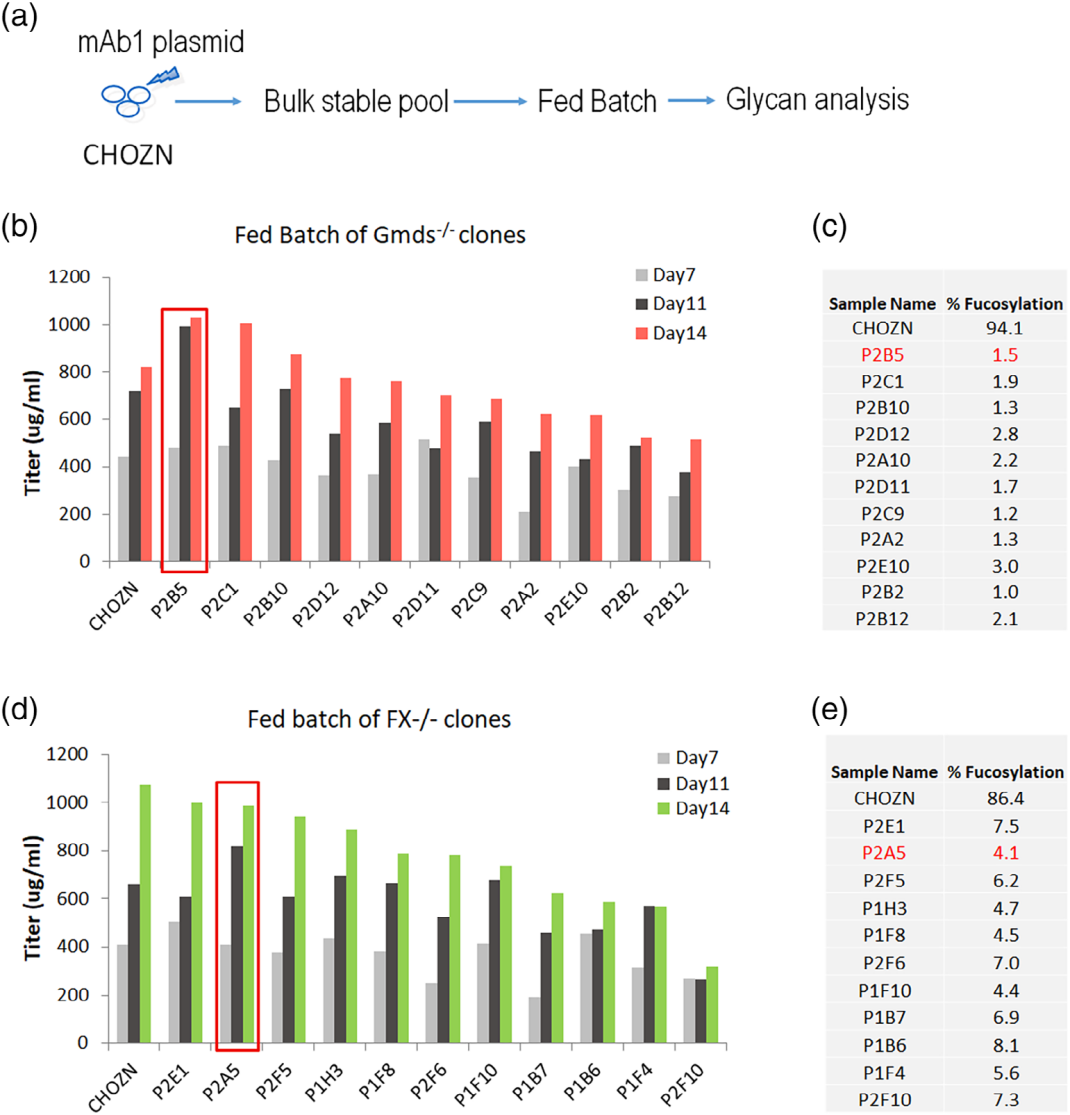
Screening and selection of top FX^−/−^ and Gmds^−/−^ clones. (a), flowchart of experiment design from transfection of mAb1‐expressing plasmid to stable pool selection, fed‐batch study and fucosylation analysis. Octet titers of mAb1 at day7, day11, and day14 of fed‐batch production for Gmds^−/−^ clones (b) and FX^−/−^ clones (d). The day14 mAbs were purified and analyzed with Gly‐Q for fucosylation levels in Gmds^−/−^ clones (c) and FX^−/−^ clones (e). Red rectangles highlighted the final lead clones. Gmds, GDP mannose‐4,6‐dehydratase

A total of 12 FX^−/−^ clones and 11 Gmds^−/−^ clones have been confirmed based on three lines of data, that is, the genomic nonsense mutations, the loss of expected proteins and the functional disruptions of fucosylation pathway. To test if the KO cell lines are suitable to express afucosylated mAb, we used a proprietary IgG1, mAb1, as the protein of interest to generate bulk pools from each of the 23 host cell lines. Except for one FX^−/−^ clone, P1B10, that did not survive the MSX selection and no pool was generated for that cell line, mAb1 expressing Bulk pools were obtained from the rest of 11 FX^−/−^ clones and 11 Gmds^−/−^ clones. A 14‐day fed‐batch production was performed from the pools, and their titers were determined by Octet, compared, and ranked (Figure 3b,d). Compared to parental CHOZN cells that led to 0.8 to 1.1 g/L of mAb1 at day14, four FX^−/−^ host clones (P1H3, P2A5, P2E1, and P2F5) and four Gmds^−/−^ host clones (P1A10, P1B10, P2B5, and P2C1) generated similar levels of mAb1 titers ranging from 0.8 to 1.0 g/L.

Next, we performed a glycan profile analysis of mAb1 from the pool production. N‐linked Glycans were released by PNGaseF digestion of each mAb1 that was purified via Protein A affinity column. The released glycans were analyzed by capillary electrophoresis (CE) on Gly‐Q. In contrast to 86.4%–94.1% of fucosylated glycan from CHOZN cells, only 6.3%–8.1% of fucosylated glycan was observed from the 11 FX^−/−^ clones and 1.0–3.0% from the 11 Gmds^−/−^ clones (Figure 3c,e).

We narrowed down the engineered clones to two top‐performing clones for each target based on the data from cell line growth robustness, capability for MSX selection tolerance, cell line long‐term stability, production titers, and afucosylation levels of mAb1 generated from the pools (Supplement data). Clone P2A5 was selected as the lead host clone and P2E1 as a backup for FX^−/−^; Clone P2B5 was the lead for Gmds^−/−^ and P2C1 was the backup. From our data, FX^−/−^ and Gmds^−/−^ CHO clones had robust cell growth, and their production titers were comparable to CHOZN cells. Both KO cell lines have the potential to be utilized for the manufacturing of afucosylated mAbs for clinical use.

### 3.3 mAbs produced from FX ^−/−^ hosts carry new glycan modifications

**FIGURE 4:**
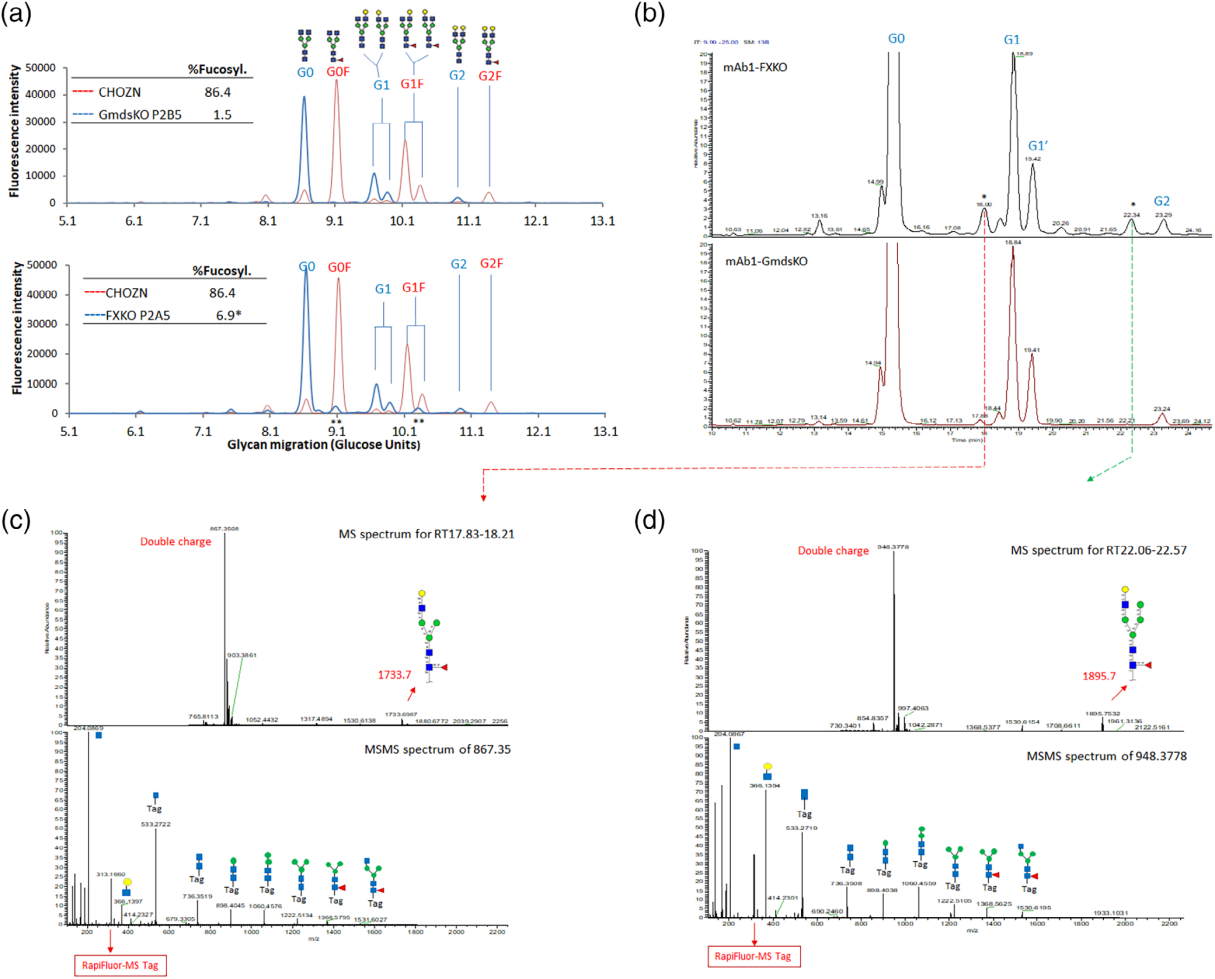
Identification of novel glycans in FX^−/−^ clones. (a), Gly‐Q analysis of the N‐Glycan profiles of mAb1 expressed in CHOZN, FX^−/−^ clone P2A5 and Gmds^−/−^ clone P2B5. Major glycans are listed as G0, G1, G2, G0F, G1F, G2F. 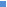 mannose; 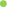 N‐Acetylglucosamine (GlcNac); 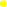 galactose; 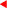 fucose. ** two novel glycan peaks. (b), Liquid Chromatograph of mAb1 glycan sample from FX^−/−^ clone P2A5 and Gmds^−/−^ clone P2B5. (c–d), Fluorescence/mass spectrometry (LC‐FLR‐MS). The two glycan peaks uniquely observed in FX^−/−^ clones were analyzed for Mass spectrum. The doubly charged peaks were further labeled with RapiFluo mass tag for another Mass spectrum analysis. Gmds, GDP mannose‐4,6‐dehydratase. Gmds, GDP mannose‐4,6‐dehydratase; mAb, marketed therapeutic antibodies

From the 11 FX^−/−^ hosts, the glycans of the expressed mAb1 were not completely afucosylated. 6%–8% of the glycans were fucosylated and that was too significant to be ignored (Figure 4a, bottom panel). Two unique glycan fraction/peaks with glucose unit (GU) of 9.07 and 10.31, were very close to but not identical to G0F with 9.12 GU and G1F(α1,6) with 10.16 GU and G1F(α1,3) with 10.37 GU. The unique peaks were not observed in mAb1 from any of the Gmds^−/−^ hosts. To further characterize the unusual glycan peaks in FX^−/−^ hosts, we analyzed the samples with liquid chromatograph/fluorescence/ mass spectrometry technology (LC‐FLR‐MS). In the LC analysis, the Glycan of 9.07GU had a retention time of 18.03 min, and the Glycan of 10.31GU had a retention time of 22.34 min (Figure 4b). The 18.03 min peak had a mass of 1737.7 Da, and MS/MS fragmentation analysis confirmed its structure HexNAc(3)Hex(4)Fuc (1) (G1F‐GlcNAc) (Figure 4c). The 22.34 min peak had a mass of 1895.7 Da and MS/MS fragmentation analysis confirmed its structure HexNAc(3)Hex(5)Fuc (1) (Figure 4d).

### 3.4 mAbs produced from FX ^−/−^ and Gmds^−/−^ CHOZN host cell lines demonstrated enhanced FcγRIIIa binding and ADCC effects

**FIGURE 5:**
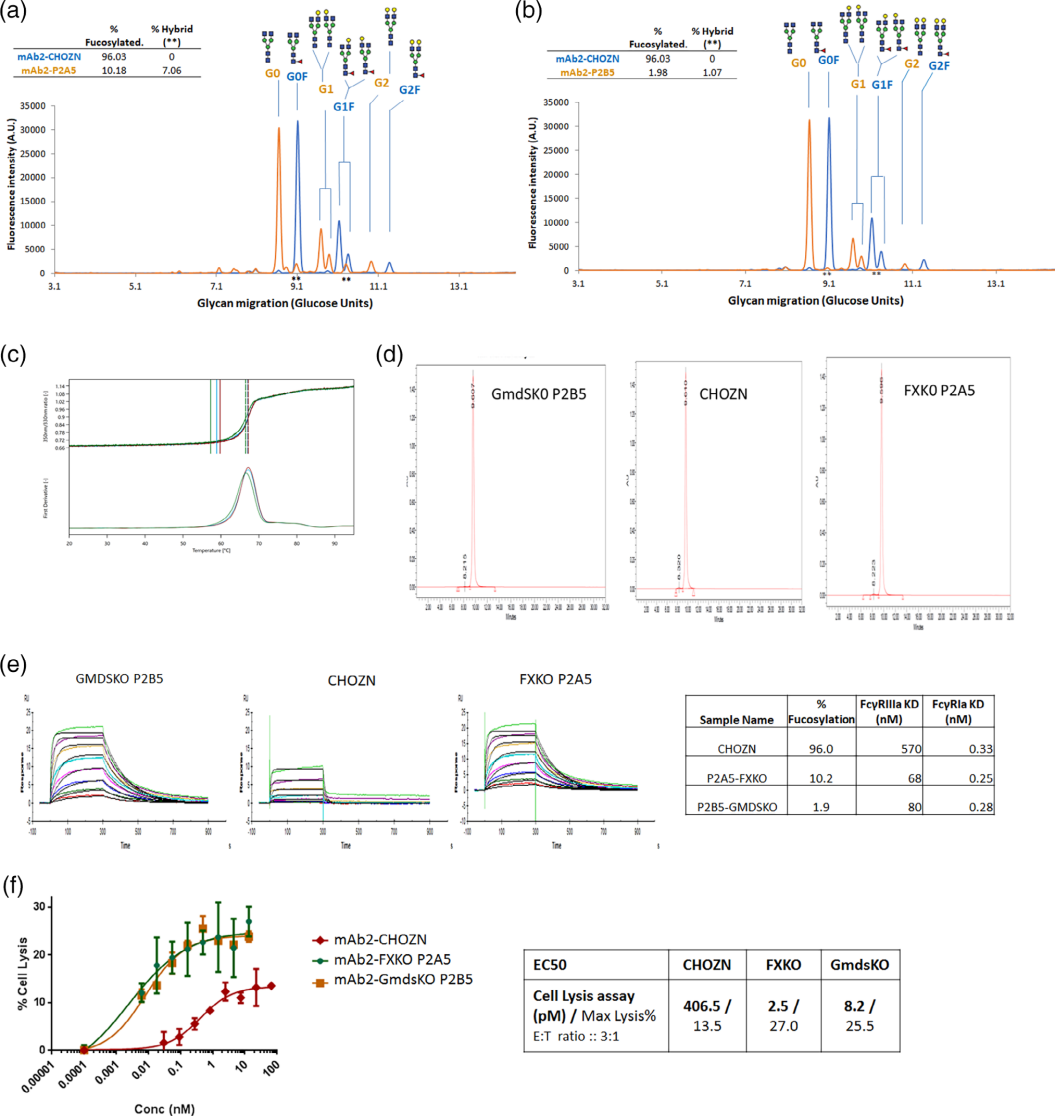
Boosted FcγRIIIa binding and ADCC efficacy for mAb2 produced from FX^−/−^ and Gmds^−/−^ CHOZN host cell lines. mAb2 was produced in CHOZN, FX^−/−^ clone P2A5 and Gmds^−/−^ clone P2B5. (a, b) glycan profiles, (c) thermal stability analysis, (d) size exclusion chromatography (SEC), (e) FcγRIIIa and FcγRIa binding assay, and (f) in vitro ADCC assay. ADCC, antibody‐dependent cellular cytotoxicity; Gmds, GDP mannose‐4,6‐dehydratase; mAb, marketed therapeutic antibodies

41TWe examined the FcγRIIIa binding and ADCC effects of a second proprietary monoclonal antibody, mAb2, produced from the two engineered cell lines. We expressed mAb2, an IgG1 with wildtype Fc domain, in the FX−/− P2A5 cells, Gmds−/− P2B5 cells, and CHOZN cells. Consistent with the fucosylation profiles of mAb1, Gly‐Q results showed that mAb2 produced from CHOZN is 96% fucosylated. In contrast, mAb2 from FX−/− P2A5 cells is 10.1% fucosylated including 3.4% of G1F‐GlcNAc and 3.7% of HexNAc(3)Hex(5)Fuc (1), and mAb2 from Gmds−/− P2B5 is 1.9% fucosylated (Figure 5a,b). mAb2 with these glycan modifications demonstrated no difference in thermal stability by nano differential scanning fluorimetry (DSF) and molecular size by size exclusion chromatography (Figure 5c,d).

Surface plasmon resonance (SPR) binding assays by Biacore showed that the disassociation constant (Equilibrium dissociation constant [KD]) of mAb2 from CHOZN, FX−/− P2A5, and Gmds−/− P2B5 cells binding to FcγRIIIa were 570 nM, 80 nM, and 68 nM, to FcγRIa were 0.33 nM, 0.25 nM, and 0.28 nM respectively (Figure 5e). These data suggested that the afucosylated mAb2 produced from both FX−/− and Gmds−/−cells had significant increased binding to FcγRIIIa but had no effect on the bindings to FcγRIa.

We next examined how ADCC effects were affected by the afucosylated mAb with the increased FcγRIIIa bindings. Cells expressing corresponding cell surface antigen for mAb2 were preincubated with serial dilutions of mAb2 followed by exposure to purified human NK cells to induce ADCC‐mediated cell lysis. The maximum percentage of cell lysis was 13.5% for mAb2 from CHOZN, in contrast to 27.0% from FX^−/−^ and 25.5% from Gmds^−/−^ cells (Figure 5f). The half‐maximum effective concentration (EC50) of mAb2 from CHOZN, FX^−/−^ P2A5 and Gmds^−/−^ P2B5 were 406.5pM, 2.5pM, and 8.0pM (Figure 5d). These data together suggest that the highly afucosylated mAb2 generated from FX^−/−^ and Gmds^−/−^ CHO cells both had much higher ADCC effects (~50–160 fold). The 90% of afucosylated mAb2 from FX^−/−^ and 98% afucosylated mAb2 from Gmds^−/−^ have no significant difference in FcγRIIIa bindings and in ADCC effects, which indicated either KO cell line could be used as a master host to produce afucosylated mAbs.

These results also demonstrated that the hybrid glycans, G1F‐GlcNAc and HexNAc(3)Hex(5)Fuc (1), found on mAbs expressed from FX^−/−^ CHO cells did not interfere in FcγRIIIa binding and ADCC effects. These novel glycans are not distinguished enough to become critical attributes that affect either biophysics or function of therapeutic mAb in vitro.

## 4 DISCUSSION

CHOK1SV‐GS^−/−^ and CHOK1‐GS^−/−^ cells (CHOZN) have their endogenous GS gene disrupted and have been widely used for the manufacturing of biotherapeutics, owing to its stringent MSX selection and superior productivity.^22^ Gene modifications on these host cells have generated more desired host cell lines.^23^, ^24^, ^25^ To modify the protein fucosylation pathway and take advantage of the CHOZN superior genetic background, we edited Gmds and FX genomic loci in CHOZN cells and obtained KO cell lines for each of the genes. The top engineered host clones all grow similarly as CHOZN cells during routine cell passaging, in bulk transfection and MSX selection study. In the fed‐batch production study with the use of bulk pools, the engineered clones produced up to 1 g/L mAbs similar to what CHOZN cells can generate. Loss of either gene led to highly afucosylated N‐linked glycans on tested mAbs, and because of that, both the FcγRIIIa binding and in vitro ADCC effects of the afucosylated mAb were dramatically boosted.

In this study, we performed head‐to‐head comparisons of Gmds^−/−^ CHOZN and FX^−/−^ CHOZN cells, which were generated in the cells of the same genetic background. Previous proof‐of‐concept studies have demonstrated Gmds^−/−^ DG44 cells can be used as a host to produce afucosylated mAbs.^19^ However, the Gmds^−/−^ DG44 cells were generated with the use of traditional homologous recombination, which has extremely low efficiency and the technology itself could not provide large numbers of KO host cells for screening. Primarily because of that reason and the DG44 less favorable genetic background, the mAb productivity of the top selected clone was ~500 mg/L at 14 day fed batch, which often led to a misconception that KO of the Gmds gene compromised productivity. In comparison, a recent study demonstrated that knock out of FX gene in CHO‐K1 cells generated completely afucosylated glycans with fed‐batch titers (4‐5 g/L for top clones),^15^ leaving the impression that FX^−/−^ CHO is superior to Gmds^−/−^ CHO cells. In our study, the methods to generate the KO cell lines and the standards to evaluate the cell lines are identical for both Gmds^−/−^ CHOZN and FX^−/−^ CHOZN host cells. The KO cell lines performed very similarly in characteristics such as cell growth robustness, MSX selection tolerance, cell line long‐term stability, and production titers. The only distinguishable difference is that in FX^−/−^ CHO cells, the expressed mAbs were not completely afucosylated, with 4%–10% fucosylated glycan detected, and the majority of these fucosylated glycans are novel forms (Figure 4b), as characterized by mass spectrometry. The different glycan pattern may reflect that the protein glycosylation pathway in CHO cells are not fully elucidated. Although the slight difference of fucosylation levels did not significantly affect FcγRIIIa binding and in vitro ADCC effects, the 4%–10% novel form of glycans remains to be carefully investigated if they could cause immunogenicity in vivo. Before the concern is well addressed, we consider Gmds^−/−^ CHOZN host as a better cell line for the potential clinical or commercial manufacturing of ADCC mAbs.

## CONFLICT OF INTEREST

The study was supported by Takeda Pharmaceuticals and all authors are or were Takeda employees at the time of this study.

5

### PEER REVIEW

The peer review history for this article is available at https://publons.com/publon/10.1002/btpr.3061.

## Supporting information


**Appendix** **S1**. Supporting Information.

**Appendix** **S2**. Supporting Information.
