# Supplementary material for: Generation of FX −/− and Gmds −/− CHOZN host cell lines for the production of afucosylated therapeutic antibodies
Source: Biotechnol Prog. 2020 Aug 26;37(1):e3061. doi: 10.1002/btpr.3061 (PMC7988551; doi:10.1002/btpr.3061)
Supplement: Supplementary file 1 — Appendix S1. Supporting Information. [file BTPR-37-e3061-s001.pdf]

MSX selection data and the cell growth during Fed batch were attached below for review. Some people may like to put a lot of data in the supplement; however, we value more on the short and precise presentation of data and conclusion, without distracting the majority readers who are interested in our paper.

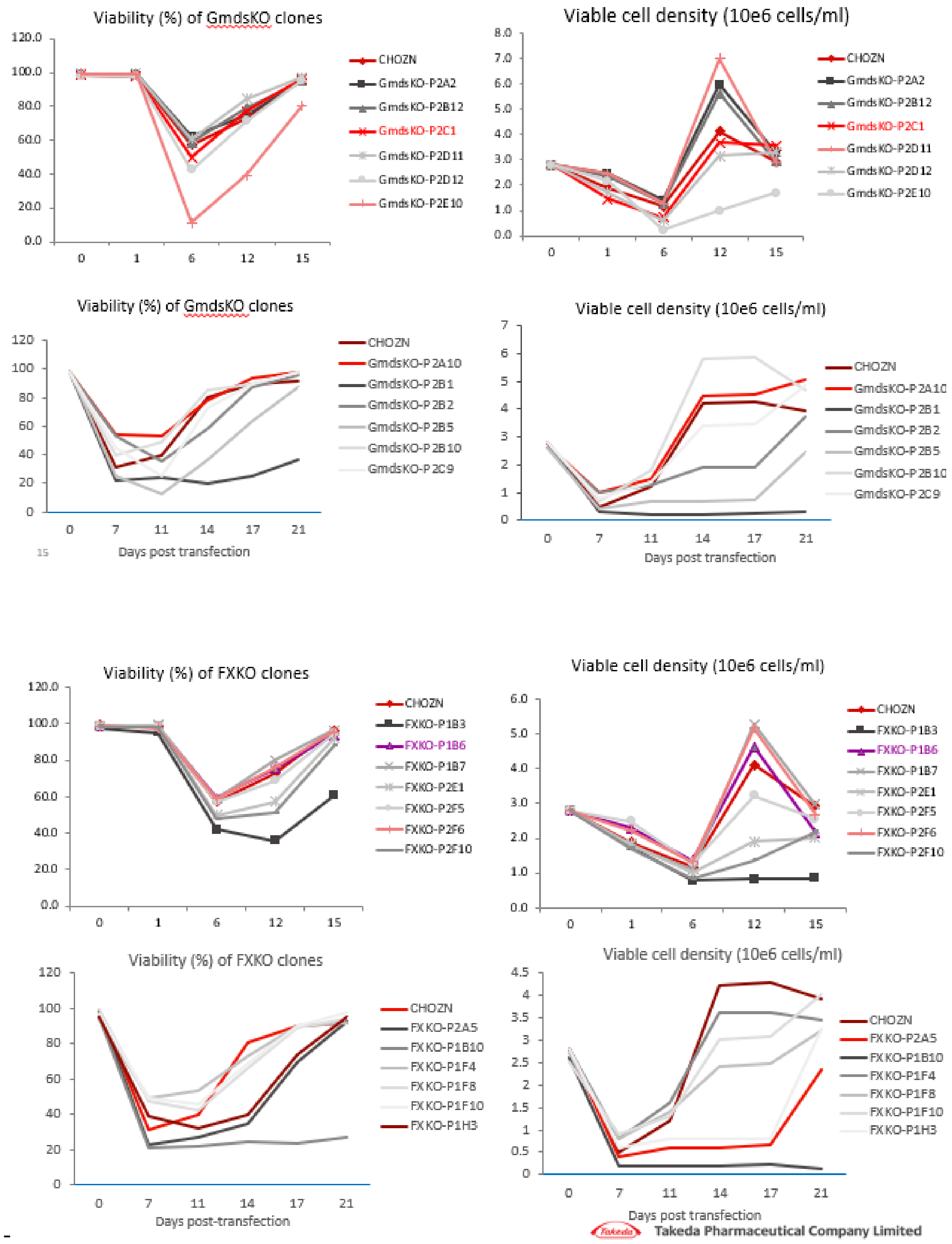

-

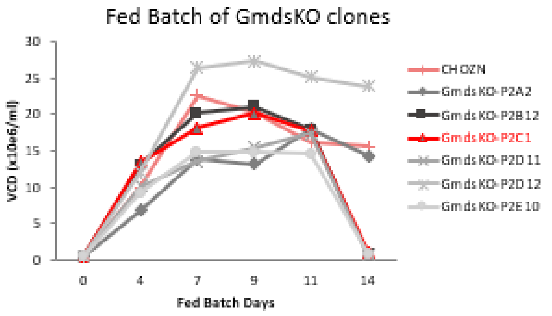
Fed Batch of GmdsKO clones
VCD (x10e6/ml)
Fed Batch Days
CHOZN
GmdsKO-P2A2
GmdsKO-P2B12
GmdsKO-P2C1
GmdsKO-P2D11
GmdsKO-P2D12
GmdsKO-P2E10

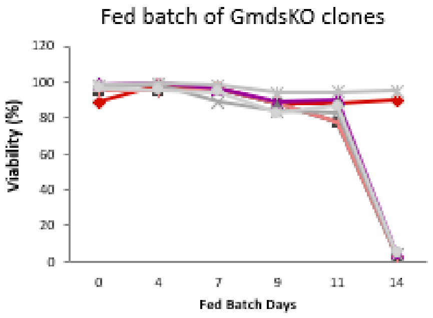
Fed batch of GmdsKO clones
Viability (%)
Fed Batch Days

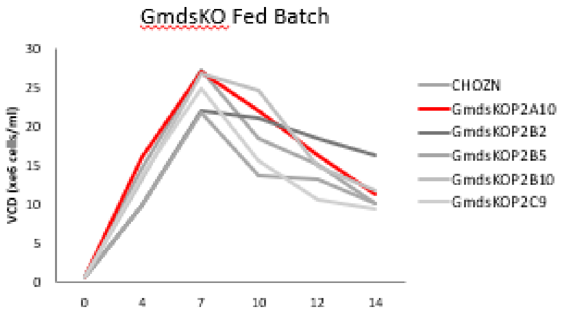
GmdsKO Fed Batch
VCD (xe6 cells/ml)
CHOZN
GmdsKOP2A10
GmdsKOP2B2
GmdsKOP2B5
GmdsKOP2B10
GmdsKOP2C9

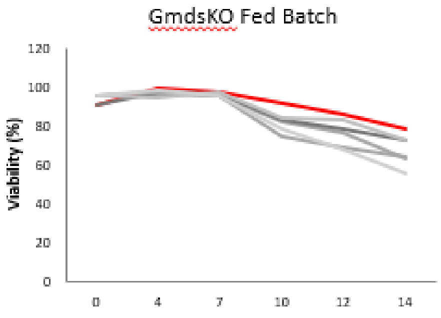
GmdsKO Fed Batch
Viability (%)

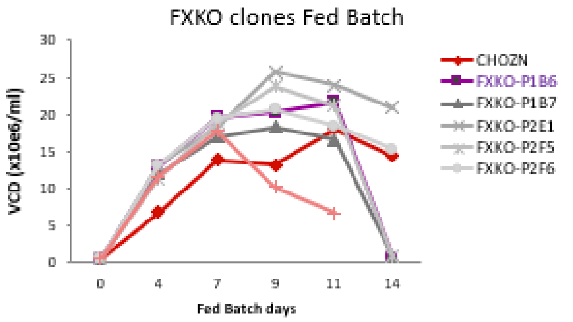
FXKO clones Fed Batch
VCD (x10e6/ml)
Fed Batch days
CHOZN
FXKO-P1B6
FXKO-P1B7
FXKO-P2E1
FXKO-P2F5
FXKO-P2F6

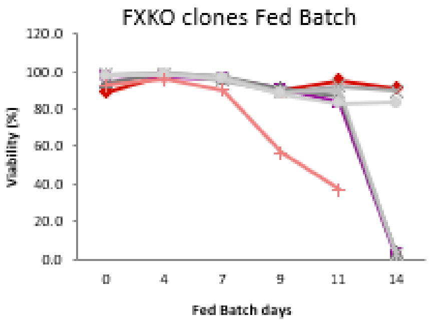
FXKO clones Fed Batch
Viability (%)
Fed Batch days

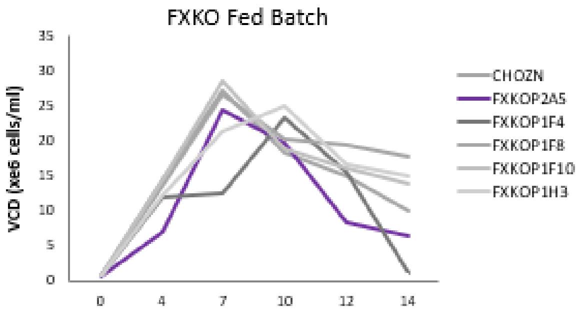
FXKO Fed Batch
VCD (xe6 cells/ml)
CHOZN
FXKOP2A5
FXKOP1F4
FXKOP1F8
FXKOP1F10
FXKOP1H3

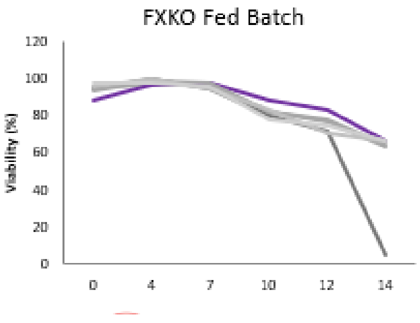
FXKO Fed Batch
Viability (%)
